# A retrospective population-based study of childhood hospital admissions with record linkage to a birth defects registry

**DOI:** 10.1186/1471-2431-9-32

**Published:** 2009-05-10

**Authors:** Lyn Colvin, Carol Bower

**Affiliations:** 1 Telethon Institute for Child Health Research, Centre for Child Health Research, The University of Western Australia, Perth, WA, Australia

## Abstract

**Background:**

Using population-based linked records of births, deaths, birth defects and hospital admissions for children born 1980–1999 enables profiles of hospital morbidity to be created for each child.

**Methods:**

This is an analysis of a state-based registry of birth defects linked to population-based hospital admission data. Transfers and readmissions within one day could be taken into account and treated as one episode of care for the purposes of analyses (N = 485,446 children; 742,845 non-birth admissions).

**Results:**

Children born in Western Australia from 1980–1999 with a major birth defect comprised 4.6% of live births but 12.0% of non-birth hospital admissions from 1980–2000. On average, the children with a major birth defect remained in hospital longer than the children in the comparison group for the same diagnosis. The mean and median lengths of stay (LOS) for admissions before the age of 5 years have decreased for all children since 1980. However, the mean number of admissions per child admitted has remained constant at around 3.8 admissions for children with a major birth defect and 2.2 admissions for all other children.

**Conclusion:**

To gain a true picture of the burden of hospital-based morbidity in childhood, admission records need to be linked for each child. We have been able to do this at a population level using birth defect cases ascertained by a birth defects registry. Our results showed a greater mean LOS and mean number of admissions per child admitted than previous studies. The results suggest there may be an opportunity for the children with a major birth defect to be monitored and seen earlier in the primary care setting for common childhood illnesses to avoid hospitalisation or reduce the LOS.

## Background

Birth defects are a major source of infant and child morbidity and mortality. Each year in Western Australia (WA), birth defects (BDs) occur in 5% of live births, more than 13% of stillbirths, 40% of neonatal deaths and 37% of post-neonatal deaths.[1] The burden of hospitalisation on these children is greater than for their peers, although most studies are either based around a single hospital setting and so not population-based; or, rely on morbidity codes indicating a BD diagnosis from the hospital records. [2-4] Australian statistics provide hospitalisation data based upon the principal diagnosis at discharge from hospital and for 1999–2000, "congenital anomalies" showed 35 admissions per 1,000 children up to the age of one year, accounting for 6% of all admissions in this age group.[5] These data are based upon hospital admissions that are not linked for individuals, and not linked to BD registries. As the data are only based on discharges where the diagnosis is coded as a congenital anomaly, they do not provide information on all hospital inpatient morbidity for the children with BDs nor how it is changing over time.

The aim of this study was to conduct a record linkage of population-based birth defects registry and hospital discharge data to demonstrate the true burden of hospitalisation for children with BDs and to compare admission rates and lengths of stay for common hospital admissions for children with and without a major birth defect.

## Methods

This was a population-based historical cohort study of administrative data relating to all live births in WA from 1980 to 1999 inclusive (N = 485,446) and hospital discharges from 1980–2000 inclusive. The WA Data Linkage System (WADLS) uses the Automatch software package with probabilistic matching based upon medical record number, surname, first given name, initial, date of birth, sex and address as the principal matching fields. Surnames are changed to a coded format in order to overcome the effects of most discrepancies in the spelling. First the NYSIIS (New York State Intelligence Information System) name compression algorithm is applied.[6] This carries out such tasks as bringing together commonly confused letter groups like 'ch' and 'gh' or 'sh' and 'sch' as well as removing vowels. The surnames are then Soundexed.[7], which involves giving the same code to similar sounding non-initial constants. The resulting compression and Soundex codes are assigned different weights for agreement depending upon their frequency in the population. Clerical checking of additional information is undertaken for possible matches that fall between definite matches and non-definite matches. Missed links have been estimated at 0.11%.[8] According to Australian Census statistics, permanent migration out of WA in 2001 was 2.7% of the population.[9] The WADLS has been validated and has been used extensively for health research. [10-14] De-identified data were provided from the WADLS, linking the births and deaths data from the Midwives' Notification System and the Register of Births and Deaths, to the Birth Defects Registry, and to hospital admissions data from the Hospital Morbidity Data from 1980 to 2000.

The Birth Defects Registry was established in 1980 and obtains high quality population-based information on BDs for all children born in WA.[1] Notifications to the Registry are received from over 100 sources including paediatricians, obstetricians, cytogenetics and ultrasound departments, and all genetic counselling clinics. The Registry defines a BD as a structural or functional abnormality that is present at conception or occurs before the end of pregnancy, and is diagnosed by 6 years of age. Each defect (up to 10 per case) is coded according to the 5-digit British Paediatric Association ICD-9 system and is classified as major or minor according to a method devised by the Centers for Disease Control and Prevention. Most minor defects, unless disfiguring or requiring treatment, are not recorded in the Registry. Of all cases registered, about 90% have at least one major BD. A list of exclusions can be found in the Registry's annual report.[1] Unless otherwise stated, results are for children with at least one major BD. Cases with more than one BD in a category were counted only once in each category. Those with BDs in more than one category were counted in each category. The comparison group was the population of children without a registered BD.

Comprehensive internal data validation was undertaken including the verification of date of birth, sex, birth outcome, and date and cause of death across the datasets used. The principal diagnosis for each admission to hospital is recorded on the hospital discharge summary form and is constructed from information available at the time of discharge from hospital. Discharges prior to July 1, 1999 were coded using the Ninth Revision of the International Classification of Diseases (ICD-9)[15] so discharges from July 1, 1999 to December 31, 2000 and coded using the Tenth Revision were mapped back to their equivalent ICD-9 code to allow comparisons across the admissions dataset. Validation of admission records included reviews of duplicate admission dates (N = 1,401/1,007,780; 0.14%); an admission occurring before the last discharge from hospital (N = 519; 0.05%); and, re-admissions for the same event. Records were merged into one admission record when a child was readmitted within one day of a previous discharge for the same condition (N = 7,467; 0.74%). This mostly occurred when a child was transferred between hospitals. Where admission and discharge occurred on the same day, the LOS was considered 0.5 day, to reflect some period of hospitalization (N = 84,472/384,493; 22.0%). Admissions associated with birth were excluded from all analyses. Seven admission age periods were used: up to 1 year, >1–2 years, >2–3 years, >3–4 years, >4–5 years, >5–12 years, and >12–18 years. Due to the cut-off point of 2000 for hospital admissions (dictated by the ethics approval for the study), complete years of follow up were not available for all children in all age periods; for example, admissions for a child born in 1993 would only be included up to their 5^th ^birthday in 1998 as they did not reach the age of 12 years by 2000 in order to be included in the 5–12 years age cohort.

The denominators for the calculation of admissions per 1,000 children were the number of children alive at the beginning of the age period and the numerators were the number of children admitted. In order to adjust for the varying survival rates over the study period, results were stratified by age group and included only the children alive at the start of the age period. Child admission rates were calculated using rate ratios (RR), adjusting for year of birth. To assess the overall burden of hospitalization for children with a major BD up to the age of five years, specific BDs were analysed to find those with the highest number of admissions, the longest mean LOS for one admission, the greatest number of admissions per child, and the longest mean total LOS (summed over all admissions).

Data validation and analyses were undertaken using SAS version 9.1.[16] the SAS GENMOD procedure was used to calculate RR and 95% confidence intervals; the SAS MEANS procedure to assess differences in means; and the NPAR1WAY procedure to assess differences in medians. The researchers received all data in a de-identified form from the WADLS. The person-based linkage was approved by the ethics and confidentiality committee of the Health Department of WA and permission to use the required data was obtained from the relevant data custodians.

## Results

**Figure 1:**
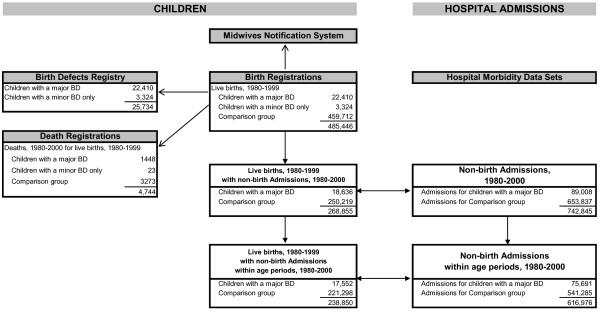
**Flow chart of data linkage for study population**.

There were 25,734 live born children registered with a BD from 1980 to 1999 out of a total population of 485,446 live births (5.3%). Of the children with BDs, 87.1% had at least one major BD. Of the children with a major BD, 10.5% (N = 2,364) had more than two defects reported. Half of all children with a major BD belonged to only two diagnostic categories: 29.2% (N = 6,546) with uro-genital defects only; 20.2% (N = 4,516) with musculo-skeletal defects only (139 (0.6%) children had defects in both categories). Excluding records of birth admissions and children with only a minor BD, and after validation of the data, 49.5% (238,850/482,122) of all children had a record of at least one hospital admission from 1980 to 2000, with 616,976 hospital admission records available for analyses within the seven age periods. A flow chart of the study population selection is found in Figure 1.

### Hospital admissions of children with a major birth defect compared with all other live births

Around half (54.4%, N = 250,219) of the children in the comparison group were admitted to hospital, compared to 83.2% (N = 18,636) of the BDs group (*P *< 0.0001). Admissions for children with a BD accounted for 12.0% (N = 89,008/742,845) of all admissions. In both groups, males were more likely to be admitted: 59.1% males and 49.6% females were admitted within the comparison group; 88.8% males and 74.8% females within the BDs group (*P *< 0.0001). A child with a major BD was more likely to be admitted to hospital in all age periods and, in particular, excluding their birth admission, 2.5 times more likely to be admitted to hospital in each of the first 5 years of life than the comparison group (Table 1). In all age groups, the mean annual LOS for the BD group was highly significantly greater than the mean annual LOS for the comparison group (*P *< 0.0001). The median LOS for the BD group was also highly significantly greater than the median LOS for the comparison group up to 5 years of age (2.0 days vs. 1.0 days).

**Table 1:** Non-birth Admissions, births 1980–1999

	Children with a Major Birth Defect				Children without a Birth Defect (Comparison Group)				
			
Age Period	No. Children at start of age period	% Children Admitted^1^	Mean annual LOS per child admitted (days)	Admissions per Child Admitted	No. Children at start of age period	% Children Admitted^1^	Mean annual LOS per child admitted (days)	Admissions per Child Admitted	Rate Ratio Children Admitted (95% CI)^2^
< = 1 year	22,410	48.7	10.7	2.2	459,172	19.6	5.4	1.6	2.48 (2.45–2.52)
>1–2 years	20,057	36.8	6.6	1.9	433,341	14.9	4.1	1.5	2.46 (2.42–2.51)
>2–3 years	18,777	28.9	5.1	1.8	409,084	11.4	2.9	1.4	2.53 (2.47–2.59)
>3–4 years	17,456	25.5	4.6	1.7	385,509	10.1	2.5	1.3	2.52 (2.45–2.59)
>4–5 years	16,124	23.0	4.2	1.6	361,696	9.3	2.3	1.3	2.46 (2.39–2.54)
>5–12 years	7,983	51.1	1.2	3.0	195,409	31.7	0.5	1.8	1.61 (1.58–1.65)
>12–18 years	2,337	40.9	1.6	2.8	61,447	33.6	0.8	1.9	1.22 (1.16–1.28)

^1 ^age group >5–12 yrs spans 7 years, >12–18 yrs spans 6 years. All other age groups included 1 year of follow up.

^2 ^adjusted for year of birth

### Hospital admissions within major birth defect categories

In every age group, admission rates per 1,000 children alive at the start of the age period for every category of major BD were considerably higher than for the comparison children in the corresponding age group (Table 2). In all age groups, children in every BD category had longer mean LOS and mean annual LOS, compared with children without BDs (Table 3).

**Table 2:** Number of children admitted per year, per 1000 children alive at the beginning of the age period, by diagnostic category and age period, births 1980–1999

	Age Period						
	
Diagnostic Category^1^	< = 1 year	>1–2 yrs	>2–3 yrs	>3–4 yrs	>4–5 yrs	>5–12 yrs	>12–18 yrs
Cardiovascular Defects	1,370	860	592	495	424	232	147
Chromosome Defects	1,282	1,252	919	703	780	411	256
CA of Ear, Face and Neck	1,078	704	455	388	481	249	167
CA of Eye	1,802	1,165	875	700	641	345	203
CA of Integument	962	652	437	437	416	282	193
Gastro-Intestinal Defects	1,800	802	546	439	386	252	165
Musculo-Skeletal Defects	830	555	385	323	300	184	174
Nervous System Defects	1,474	1,363	993	880	818	516	399
Respiratory System Defects	1,231	1,371	1,047	984	873	374	256
Uro-Genital Defects	863	741	543	430	356	206	158
Other Major Defects^2^	1,506	1,141	907	770	651	463	451

Any Major Birth Defect	1,052	705	510	424	371	221	190
Comparison Group	307	219	155	130	117	81	104

^1 ^A child may be in more than one diagnostic category of major birth defects

^2 ^These include: other and unspecified congenital anomalies, cystic fibrosis, hereditary haemolytic anaemias, congenital hypothyroidism, coagulation defects, neurofibromatosis, and muscular dystrophies and other myopathies

CA = congenital anomalies

**Table 3:** Mean LOS for a single admission in days per child (and mean annual total LOS in days per child), by diagnostic category and age period, births 1980–1999

	Age Period						
	
Diagnostic Category^1^	Mean LOS (Mean annual LOS)						
	
	< = 1 year	> 1–2 yrs	> 2–3 yrs	> 3–4 yrs	> 4–5 yrs	> 5–12 yrs	> 12–18 yrs
Cardiovascular Defects	6.0 (15.4)	3.7 (8.0)	3.0 (5.9)	3.2 (5.6)	2.4 (4.1)	2.4 (1.1)	3.3 (1.3)
Chromosome Defects	6.3 (15.2)	3.7 (9.5)	2.8 (6.6)	2.7 (5.1)	2.0 (3.8)	2.3 (1.3)	2.1 (0.8)
CA of Ear, Face and Neck	4.8 (10.6)	4.0 (7.1)	1.8 (2.9)	1.9 (3.1)	2.8 (4.5)	2.5 (1.1)	3.1 (1.1)
CA of Eye	4.8 (13.6)	2.6 (5.7)	2.4 (4.9)	2.1 (3.7)	2.5 (4.2)	2.5 (1.3)	2.7 (1.2)
CA of Integument	4.0 (8.0)	2.1 (4.3)	1.8 (2.9)	1.6 (2.7)	2.0 (3.1)	1.8 (1.0)	4.7 (2.0)
Gastro-Intestinal Defects	6.0 (13.3)	4.1 (8.4)	3.1 (5.7)	2.9 (4.7)	2.5 (4.0)	2.9 (1.4)	2.7 (1.1)
Musculo-Skeletal Defects	4.9 (10.7)	3.7 (7.7)	3.3 (5.9)	2.9 (4.9)	2.9 (4.9)	3.0 (1.3)	4.0 (1.7)
Nervous System Defects	6.7 (19.4)	4.8 (12.7)	4.6 (10.5)	4.2 (8.5)	4.1 (8.9)	4.0 (3.0)	5.0 (3.8)
Respiratory System Defects	7.2 (25.4)	4.3 (13.4)	3.3 (8.7)	3.3 (7.2)	1.9 (4.5)	2.2 (1.4)	1.6 (0.7)
Uro-Genital Defects	4.3 (8.3)	3.1 (5.0)	2.7 (4.2)	2.6 (4.0)	2.7 (3.9)	2.5 (0.9)	2.5 (1.0)
Other Major Defects^2^	6.3 (17.7)	3.9 (10.2)	3.3 (7.7)	3.3 (7.1)	3.0 (6.2)	3.5 (2.5)	5.1 (4.6)

Any Major Birth Defect	5.0 (10.7)	3.4 (6.6)	2.9 (5.1)	2.8 (4.6)	2.6 (4.2)	2.8 (8.4)	3.5 (9.7)
Comparison Group	3.5 (5.5)	2.8 (4.2)	2.2 (3.0)	1.9 (2.5)	1.8 (2.4)	2.1 (0.5)	2.4 (0.8)

^1 ^A child may be in more than one diagnostic category of major birth defects

^2 ^These include: other and unspecified congenital anomalies, cystic fibrosis, hereditary haemolytic anaemias, congenital hypothyroidism, coagulation defects, neurofibromatosis, and muscular dystrophies and other myopathies

CA = congenital anomalies

### Leading causes of hospital admission up to 5 years of age

The five most common causes for admission to hospital for the comparison group, for major BDs overall, and within each BD category were summarised using the first three digits of the ICD-9 code assigned as the principal diagnosis at hospital discharge (Table 4). Asthma (ICD-9 code 493) was the leading cause of admission in the comparison group and the second most common cause of admission for all major BDs. Asthma was one of the top five causes for admission in all but three of the 11 BD categories (chromosome defects, nervous system defects and respiratory system defects). The mean LOS of 2.5 days for asthma admissions in the BDs group was significantly longer than the mean of 2.2 days for the comparison group: *P *< 0.0001. Conditions related to BDs were the leading cause of admission in all categories of BDs.

**Table 4:** Five leading causes of admission to hospital up to 5 years of age, by diagnostic category, births 1980–1995

Diagnostic Category^1^	ICD-9	Prinicipal Diagnosis	No. Adm	*(% Adm)*	No. Chn Adm	*(% Chn Adm)*	Adm/Ch Adm	Mean LOS
**Cardiovascular Defects**	745	Bulbus cordis anomalies and anomalies of cardiac septal closure	1,226	*(13.2)*	635	*(9.7)*	1.9	5.5
9,259 admissions	747	Other congenital anomalies of circulatory system	491	*(5.3)*	363	*(5.6)*	1.4	5.8
2,010 children admitted	746	Other congenital anomalies of heart	408	*(4.4)*	266	*(4.1)*	1.5	5.4
	465	Acute upper respiratory infections of multiple or unspecified sites	368	*(4.0)*	233	*(3.6)*	1.6	4.0
	493	Asthma	367	*(4.0)*	164	*(2.5)*	2.2	2.8

**Chromosome Defects**	381	Nonsuppurative otitis media and Eustachian tube disorders	213	*(7.2)*	135	*(6.7)*	1.6	0.6
2,964 admissions	745	Bulbus cordis anomalies and anomalies of cardiac septal closure	156	*(5.3)*	82	*(4.1)*	1.9	4.9
528 children admitted	519	Other diseases of respiratory system	153	*(5.2)*	72	*(3.6)*	2.1	5.8
	465	Acute upper respiratory infections of multiple or unspecified sites	146	*(4.9)*	81	*(4.0)*	1.8	4.5
	464	Acute laryngitis and tracheitis	138	*(4.7)*	81	*(4.0)*	1.7	2.5

**CA of Ear, Face and Neck**	744	Congenital anomalies of ear, face and neck	84	*(16.8)*	72	*(18.4)*	1.2	1.8
500 admissions	465	Acute upper respiratory infections of multiple or unspecified sites	30	*(6.0)*	11	*(2.8)*	2.7	6.2
139 children admitted	381	Nonsuppurative otitis media and Eustachian tube disorders	28	*(5.6)*	23	*(5.9)*	1.2	0.6
	493	Asthma	15	*(3.0)*	7	*(1.8)*	2.1	5.4
	550	Inguinal hernia	15	*(3.0)*	13	*(3.3)*	1.2	2.7

**CA of Eye**	743	Congenital anomalies of eye	496	*(24.8)*	183	*(14.5)*	2.7	2.3
2,001 admissions	381	Nonsuppurative otitis media and Eustachian tube disorders	79	*(4.0)*	53	*(4.2)*	1.5	0.8
356 children admitted	493	Asthma	61	*(3.1)*	24	*(1.9)*	2.5	2.8
	465	Acute upper respiratory infections of multiple or unspecified sites	57	*(2.9)*	38	*(3.0)*	1.5	5.9
	783	Symptoms concerning nutrition, metabolism and development	47	*(2.4)*	31	*(2.5)*	1.5	6.4

**CA of Integument**	228	Haemangioma and lymphangioma, any site	266	*(13.8)*	188	*(14.3)*	1.4	3.1
1,935 admissions	757	Congenital anomalies of the integument	241	*(12.5)*	83	*(6.3)*	2.9	0.8
546 children admitted	216	Benign neoplasm of skin	84	*(4.3)*	59	*(4.5)*	1.4	3.3
	493	Asthma	71	*(3.7)*	39	*(3.0)*	1.8	2.2
	780	General symptoms	65	*(3.4)*	43	*(3.3)*	1.5	2.1

**Gastro-Intestinal Defects**	749	Cleft palate and cleft lip	922	*(11.2)*	557	*(9.3)*	1.7	8.8
8,205 admissions	750	Other congenital anomalies of upper alimentary tract	873	*(10.6)*	766	*(12.8)*	1.1	4.8
1,947 children admitted	381	Nonsuppurative otitis media and Eustachian tube disorders	496	*(6.1)*	342	*(5.7)*	1.5	0.7
	751	Other congenital anomalies of digestive system	439	*(5.4)*	284	*(4.7)*	1.5	9.3
	493	Asthma	283	*(3.5)*	123	*(2.1)*	2.3	2.4

**Musculo-Skeletal Defects**	754	Certain congenital musculoskeletal deformities	1,140	*(11.3)*	656	*(9.1)*	1.7	3.6
10,049 admissions	V54	Other orthopaedic aftercare	420	*(4.2)*	270	*(3.8)*	1.6	1.4
2,610 children admitted	493	Asthma	368	*(3.7)*	180	*(2.5)*	2.0	2.7
	756	Other congenital musculoskeletal anomalies	352	*(3.5)*	263	*(3.7)*	1.3	6.2
	381	Nonsuppurative otitis media and Eustachian tube disorders	326	*(3.2)*	235	*(3.3)*	1.4	0.9

**Nervous System Defects**	742	Other congenital anomalies of nervous system	311	*(6.3)*	190	*(5.8)*	1.6	6.4
4,968 admissions	996	Complications peculiar to certain specified procedures	252	*(5.1)*	115	*(3.5)*	2.2	8.9
786 children admitted	780	General symptoms	227	*(4.6)*	139	*(4.3)*	1.6	3.7
	345	Epilepsy	220	*(4.4)*	70	*(2.2)*	3.1	10.2
	741	Spina bifida	168	*(3.4)*	102	*(3.1)*	1.6	7.6

**Respiratory System Defects**	748	Congenital anomalies of respiratory system	84	*(10.0)*	45	*(9.0)*	1.9	7.4
838 admissions	519	Other diseases of respiratory system	72	*(8.6)*	25	*(5.0)*	2.9	8.2
117 children admitted	465	Acute upper respiratory infections of multiple or unspecified sites	40	*(4.8)*	17	*(3.4)*	2.4	6.5
	464	Acute laryngitis and tracheitis	34	*(4.1)*	16	*(3.2)*	2.1	1.5
	381	Nonsuppurative otitis media and Eustachian tube disorders	34	*(4.1)*	19	*(3.8)*	1.8	1.2

**Uro-Genital Defects**	752	Congenital anomalies of genital organs	3,524	*(22.2)*	2,879	*(23.3)*	1.2	2.5
15,855 admissions	599	Other disorders of urethra and urinary tract	675	*(4.3)*	449	*(3.6)*	1.5	4.4
4,959 children admitted	593	Other disorders of kidney and ureter	671	*(4.2)*	510	*(4.1)*	1.3	5.6
	493	Asthma	613	*(3.9)*	296	*(2.4)*	2.1	2.2
	550	Inguinal hernia	457	*(2.9)*	422	*(3.4)*	1.1	1.6

**Other Major Defects**^2^	277	Other and unspecified disorders of metabolism	319	*(3.3)*	110	*(1.9)*	2.9	11.4
9,575 admissions	519	Other diseases of respiratory system	313	*(3.3)*	157	*(2.7)*	2.0	7.9
1,665 children admitted	493	Asthma	287	*(3.0)*	130	*(2.2)*	2.2	3.1
	780	General symptoms	281	*(2.9)*	186	*(3.2)*	1.5	3.5
	783	Symptoms concerning nutrition, metabolism and development	274	*(2.9)*	185	*(3.2)*	1.5	6.2

**All Major Birth Defects**	752	Congenital anomalies of genital organs	3,568	*(7.1)*	2,916	*(8.0)*	1.2	2.5
50,432 admissions	493	Asthma	1,903	*(3.8)*	883	*(2.4)*	2.2	2.5
13,368 children admitted	381	Nonsuppurative otitis media and Eustachian tube disorders	1,594	*(3.2)*	1,152	*(3.2)*	1.4	0.8
	465	Acute upper respiratory infections of multiple or unspecified sites	1,457	*(2.9)*	904	*(2.5)*	1.6	2.6
	780	General symptoms	1,278	*(2.5)*	1,032	*(2.8)*	1.2	3.7

**Comparison Group**	493	Asthma	27,584	*(8.3)*	14,229	*(5.1)*	1.9	2.2
334,061 admissions	780	General symptoms	16,930	*(5.1)*	13,396	*(4.8)*	1.3	2.0
154,517 children admitted	V65	Other persons seeking consultation without complaint or sickness	13,930	*(4.2)*	11,549	*(4.1)*	1.2	3.3
	466	Acute bronchitis and bronchiolitis	13,913	*(4.2)*	11,058	*(4.0)*	1.3	3.9
	464	Acute laryngitis and tracheitis	12,192	*(3.6)*	10,064	*(3.6)*	1.2	1.8

^1 ^A child may be in more than one diagnostic category of major birth defects

^2 ^These include: other and unspecified congenital anomalies, cystic fibrosis, hereditary haemolytic anaemias, congenital hypothyroidism, coagulation defects, neurofibromatosis, and muscular dystrophies and other myopathies

CA = congenital anomalies

The second most common reason for admission in the comparison group was for "780 general symptoms" and again the mean LOS was significantly longer for admissions for this reason in the BDs group: 3.7 days cf. 2.0 days, *P *< 0.0001. The mean LOS for the third most common reason for admission in the control group, "V65 other persons seeking consultation without complaint or sickness," was not significantly different to the mean LOS for the BDs group. These children were predominantly from remote areas of the state. The mean LOS for the group of ICD-9 codes, "460–466 acute respiratory infections" was significantly longer in the BDs group than for the control group: 3.7 days cf. 2.8 days, *P *< 0.0001.

### Changes in hospital admission rates up to 5 years of age

Admissions per 1,000 live births decreased over time for the comparison group but increased in all BD categories. The mean total LOS per child up to the age of five years has decreased for most diagnostic categories of major BDs as well as the comparison group for admissions from 1980 to 1999 (Table 5). The number of admissions per child admitted increased for most BD categories and decreased for the comparison group. The mean LOS decreased for all groups and the median LOS also decreased for most groups.

**Table 5:** Change in admission rates up to 5 years of age, excluding birth admissions, births 1980–1994

Birth Years	No. Chn Adm	No. Adm	Adm/1000 livebirths	Adm/Ch Adm	Mean LOS	Median LOS	Mean Total LOS/Ch Adm
**Cardiovascular Defects**							
1980–1984	528	2,412	3,327	4.6	5.1	2.0	23.2
1985–1989	626	2,763	3,313	4.4	4.9	2.0	21.6
1990–1994	721	3,524	3,589	4.9	4.2	2.0	20.4

**Chromosome Defects**							
1980–1984	124	627	3,389	5.1	5.0	3.0	25.3
1985–1989	163	832	3,870	5.1	4.4	2.0	22.5
1990–1994	198	1,277	4,783	6.4	3.6	2.0	23.0

**CA of Ear, Face and Neck**							
1980–1984	51	172	2,688	3.4	4.0	2.0	13.5
1985–1989	40	115	2,130	2.9	3.5	1.0	10.1
1990–1994	40	193	4,196	4.8	4.0	2.0	19.3

**CA of Eye**							
1980–1984	92	483	4,025	5.3	3.8	2.0	20.0
1985–1989	97	528	4,632	5.4	3.3	1.0	18.1
1990–1994	149	917	5,660	6.2	3.3	1.0	20.1

**CA of Integument**							
1980–1984	85	252	2,355	3.0	3.2	2.0	9.5
1985–1989	176	604	2,709	3.4	2.7	1.0	9.3
1990–1994	234	914	3,088	3.9	2.9	1.0	11.2

**Gastro-Intestinal Defects**							
1980–1984	546	2,262	3,534	4.1	5.8	3.0	24.0
1985–1989	643	2,648	3,703	4.1	5.2	3.0	21.4
1990–1994	630	2,566	3,629	4.1	4.2	2.0	17.1

**Musculo-Skeletal Defects**							
1980–1984	748	2,749	2,061	3.7	5.1	2.0	18.7
1985–1989	794	3,160	2,367	4.0	4.2	2.0	16.7
1990–1994	878	3,431	2,329	3.9	3.6	1.0	14.1

**Nervous System Defects**							
1980–1984	239	1,524	4,293	6.4	7.2	3.0	45.9
1985–1989	230	1,386	3,971	6.0	5.3	2.0	31.9
1990–1994	281	1,848	4,981	6.6	4.3	2.0	28.3

**Respiratory System Defects**							
1980–1984	28	221	2,483	7.9	8.0	3.0	63.1
1985–1989	36	200	2,128	5.6	4.4	1.5	24.4
1990–1994	44	385	4,583	8.8	4.3	2.0	37.6

**Uro-Genital Defects**							
1980–1984	1,172	3,628	2,573	3.1	4.4	2.0	13.6
1985–1989	1,439	4,429	2,619	3.1	3.6	2.0	11.0
1990–1994	1,946	6,410	2,935	3.3	3.1	1.0	10.1

**Other Major Defects**^1^							
1980–1984	421	2,461	4,760	5.8	5.2	2.0	30.2
1985–1989	521	2,734	4,232	5.2	5.0	2.0	26.2
1990–1994	597	3,582	4,776	6.0	4.1	1.0	24.5

**All Major Birth Defects**							
1980–1984	3,466	13,100	2,842	3.8	4.8	2.0	18.2
1985–1989	4,096	15,025	2,845	3.7	4.2	2.0	15.4
1990–1994	4,815	18,443	3,011	3.8	3.3	1.0	12.8

**Comparison Group**							
1980–1984	45,514	103,785	977	2.3	3.4	2.0	7.8
1985–1989	48,209	103,764	892	2.2	2.8	2.0	6.1
1990–1994	50,244	104,836	874	2.1	2.3	1.0	4.9

^1 ^These include: other and unspecified congenital anomalies, cystic fibrosis, hereditary haemolytic anaemias, congenital hypothyroidism, coagulation defects, neurofibromatosis, and muscular dystrophies and other myopathies

CA = congenital anomalies

### Specific birth defects with the greatest burden of hospital admission up to 5 years of age

Admissions up to 5 years of age were analysed for specific BDs (Table 6). The BD with the greatest proportion of overall admissions up to five years of age was undescended testis with 9.4% of all admissions (15.3% of all male admissions). The greatest proportion of admissions for females was for developmental dysplasia of hip (9.7%). The most number of admissions per child was for thalassaemias (50.5 admissions); 2.2 admissions for the comparison group. The longest mean LOS for a single admission was for cystic fibrosis (9.4 days); 2.8 days for the comparison group.

**Table 6:** Specific birth defects with the greatest burden of hospital admission up to 5 years of age, excluding birth admissions, births 1980–1995

**Greatest number of admissions**			
	Adm	Adm/Child Admitted	% Total Adm

Undescended Testis	6,249	2.8	9.4
Developmental Dysplasia of Hip	3,476	3.0	5.3
Ventricular Septal Defect	3,293	4.9	5.0
Vesico-Ureteric Reflux	2,506	3.7	3.8
Talipes	2,287	4.6	3.5
Pyloric Stenosis	2,013	2.8	3.0
Down Syndrome	1,924	5.6	2.9
Comparison Group	334,061	2.2	100.0
			
**Most admissions per child**			

	Adm	Adm/Child Admitted	Mean Total LOS

Thalassemias	202	50.5	29.8
Congenital Hydrocephalus (excludes those with NTD)	1,007	8.4	48.7
Tracheo-Oesophageal Fistula, Oesophageal Atresia/Stenosis	636	8.0	41.3
Microcephaly	1,051	7.8	62.5
Spina Bifida	1,158	7.0	35.9
Hirschsprung's Disease	468	6.9	49.7
Tetralogy of Fallot	781	6.9	35.4
Comparison Group	334,061	2.2	6.1
			
**Longest mean LOS (days) in one admission**			

	Adm	Adm/Child Admitted	Mean LOS

Cystic Fibrosis	576	5.2	9.4
Cystic Kidney Disease	270	4.7	8.0
Microcephaly	1,051	7.8	8.0
Hirschsprung's Disease	468	6.9	7.2
Renal Agenesis or Dysgenesis	248	6.5	6.6
Stenosis/Atresia Small Intestine	270	4.7	6.3
Encephalocoele	132	5.5	5.9
Comparison Group	334,061	2.2	2.8
			
**Longest mean total LOS (days)**			

	Adm	Adm/Child Admitted	Mean Total LOS

Microcephaly	1,051	7.8	62.5
Hirschsprung's Disease	468	6.9	49.7
Cystic Fibrosis	576	5.2	49.2
Congenital Hydrocephalus (excludes those with NTD)	1,007	8.4	48.7
Renal Agenesis or Dysgenesis	248	6.5	43.2
Tracheo-Oesophageal Fistula, Oesophageal Atresia/Stenosis	636	8.0	41.3
Cystic Kidney Disease	270	4.7	38.0
Comparison Group	334,061	2.2	6.1

* A child may have more than one major birth defect

### Leading causes of admission within the comparison group up to 5 years of age (Table 7)

**Table 7:** Leading causes of admission within the Comparison group compared with admissions for these reasons in children with a birth defect, up to 5 years of age, excluding birth admissions, births 1980–1995

		Adm N = 384,494		Children Admitted N = 167,885		Rate Ratio Children Admitted (95% CI)^1^	Mean Total LOS (days)		Median LOS (days)	
						
ICD-9	Principal Diagnosis	Major BD N = 50,432	Comparison N = 334,061	Major BD N = 13,368	Comparison N = 154,517	Major BD	Major BD	Comparison	Major BD	Comparison
493	Asthma	1,903	27,584	883	14,229	1. 30 (1.22–1.39)	5.5	4.3	2.0	2.0
780	General symptoms	1,457	16,930	1,032	13,396	1.63 (1.54–1.74)	3.7	2.6	2.0	1.0
V65	Other persons seeking consultation without complaint or sickness	683	13,930	546	11,549	1.01 (0.93–1.10)	3.8	4.0	2.0	2.0
466	Acute bronchitis and bronchiolitis	1,229	13,913	942	11,058	1.78 (1.67–1.90)	6.8	4.9	4.0	3.0
464	Acute laryngitis and tracheitis	906	12,192	643	10,064	1.36 (1.26–1.47)	2.9	2.2	1.0	1.0
009	Ill-defined intestinal infections	875	12,165	652	9,476	1.50 (1.39–1.62)	5.6	5.1	2.0	2.0
465	Acute upper respiratory infections of multiple or unspecified sites	1,278	11,994	904	9,716	1.98 (1.86–2.12)	5.2	3.4	2.0	2.0
381	Nonsuppurative otitis media and Eustachian tube disorders	1,594	11,723	1,152	9,733	2.40 (2.26–2.55)	1.1	0.8	0.5	0.5
474	Chronic disease of tonsils and adenoids	701	10,889	671	10,493	1.33 (1.24–1.44)	1.7	1.6	1.0	1.0
519	Other diseases of respiratory system	1,188	8,477	671	6,202	2.31 (2.14–2.50)	9.9	5.3	3.0	3.0
382	Suppurative and unspecified otitis media	693	8,262	568	7,069	1.68 (1.54–1.83)	2.7	2.4	1.0	1.0
521	Diseases of hard tissues of teeth	713	8,011	651	7,507	1.80 (1.67–1.95)	0.8	0.7	0.5	0.5

^1 ^adjusted for year of birth

Asthma and general symptoms were the top two reasons for admission in the comparison group up to 5 years. Children with a major BD were 1.3 times more likely to be admitted for asthma and 1.6 times more likely to be admitted for general symptoms. Children with a major BD had a mean total LOS in hospital that was one day longer than the children in the comparison group. For each of the leading 12 causes of admission within the comparison group, "V65 other persons seeking consultation without complaint or sickness" was the only one where the relative risk of admission for children with a BD was the same as for the comparison group. For all other diagnoses, children with a major BD had an increased risk of admission. The greatest RR of admission were admissions relating to 'nonsuppurative otitis media and eustachian tube disorders' (RR = 2.4), 'other diseases of the respiratory system' (RR = 2.3), and 'acute upper respiratory infections of multiple or unspecified sites' (RR = 2.0). The median LOS was similar for all children.

### Leading causes of admission for children aged 12 to 18 years

Admissions for children in the older age periods were analysed. The RR of admission was still greater for the children with a major BD (40.9% were admitted during the six year age period) compared with 33.6% of the comparison group (RR = 1.2). In this age group the leading cause for admission was for ICD-9 chapter 9, Diseases of the Digestive System (which includes 'diseases of oral cavity, salivary glands and jaws'), accounting for 19.0% of admissions in the comparison group and 14.8% in the major BD group. The next leading causes for admission in the comparison group were chapter 17, Injury and Poisoning (18.7%) and chapter 8, Diseases of the Respiratory System (10.4%). For children with a major BD, the next leading causes for admission were chapter 3, Endocrine, Nutritional and Metabolic Diseases, and Immunity Disorders (14.0%) and chapter 17, Injury and Poisoning (13.4%).

## Discussion

Our very large study of 485,446 live births provides population-based profiles of hospital admissions to enable comparisons between children with and without major BDs born over the same period in WA. Using linked administrative data for each live birth in WA from 1980–1999, hospital admissions for individual children were analysed within diagnostic categories of BDs, by age at admission and mean LOS. Net interstate migration from WA is low[9] and linkages with the deaths dataset meant the denominator data for specific age periods were more accurate. We found the admission rate up to the age of one year for a child with a major BD was 1,052 per 1,000 (1,108 per 1,000 males and 971 per 1,000 females) excluding the birth admission – three times the admission rate for children without a BD. Whilst only 4.6% of live births in WA were children with a major BD, this group accounted for 12.0% of the hospital admissions. This finding is similar to the almost 12% of paediatric hospitalisations related to birth defects and genetic diseases in California and South Carolina[4] but they are far higher than admissions for congenital anomalies (ICD-9 codes 740–759) alone in Australia ascertained by the national routinely collected statistics (35 per 1,000).[5] This occurs because, as we have shown, children with BDs are also more likely to be admitted for reasons other than a BD, and hospital discharge summaries may not always indicate that a child has a BD. Furthermore, there are also BDs classified with codes outside the ICD-9 chapter of 'Congenital Anomalies', such as thalassaemias and cystic fibrosis and these conditions can have substantial hospital morbidity. An analogous situation was observed in a study of mortality associated with birth defects[17], which demonstrated that using birth defects registry data gave a more comprehensive picture of the full burden of birth defects on mortality than was evident from cause of death statistics alone.

By excluding birth admissions, issues relating to well-baby admissions and birth circumstances can be separated from the ongoing burden of hospitalisation a child may experience. This was highlighted in the report by MacFaul and Werneke showing that large numbers of wellborn infants artificially inflate the paediatric hospital admission statistics.[18] However, to the extent that prolonged birth admissions in children with BDs and other serious perinatal conditions may occur, our data will underestimate the total burden.

Whilst admissions per 1000 children alive at the start of the age period and admissions per child admitted have decreased in the comparison group, these rates have generally increased within most categories of BD. There are several possible reasons for these trends. Many procedures are now done on an outpatient basis that formerly required hospital admission and this change may be more likely to occur for a child without a BD. New investigations and new treatment methods may require hospital admission in some children with BDs, and improved survival of children with BDs may result in increased hospitalisation. We have seen an example of this in WA in children with Down syndrome. There has been a significant increase in overall survival in these children since 1980 and a significant increase in survival for children with congenital heart disease and Down syndrome, who are very likely to require several hospital admissions.[19] There may also be an increasing tendency to admit children with BDs to hospital for preventive procedures or medication stabilization (eg changing anticonvulsant drugs, dental procedures[20]).

Mean and median LOS have decreased over time in both groups. This is probably due to the fact that many more procedures that once required a longer admission to hospital are now done as a day admission. Our analyses showed a greater mean LOS and mean number of admissions per child admitted than previous studies for children with a major BD. These children were admitted to hospital for longer even when admitted for non-BD conditions. For the most common reasons for admission in the comparison group up to 5 years of age, asthma and general symptoms, children with a BD were 30% more likely to be admitted for asthma and 60% more likely to be admitted for general symptoms. These children also remained in hospital longer than the children in the comparison group, but the median LOS was similar in both groups, indicating that some admissions in the major BD group had extended LOS. Possible reasons for this include greater complexity in treating children with BDs and identification of co-morbidities whilst in hospital that are then treated and thus delay discharge. Unfortunately, our dataset did not contain information with which we could address the potential reasons for longer stays and more admissions over time.

A 2004 report reviewed the records of 5,747 consecutive admissions to a paediatric hospital during 1996 for 4,224 children and found 71% of these children had a condition with a 'significant genetic component'.[3] Children with single-gene and chromosomal disorders had an average hospital stay (7.1 days) that was twice as long as the 3.5 days average stay for children without any pre-existing chronic medical disorder. Other similar studies have also been reported [3,4,21,22] but all of these relied upon the hospital diagnoses data for ascertaining cases of BDs and were likely to underestimate the true hospital morbidity rate per child.

The previous literature suggests that children with BDs have increased rates of hospital admission and longer stays in hospital but because of limitations in case ascertainment these may still be underestimated.

We found similar results to Viner[23] in the 12–18 years age group where females surpassed males in admissions per 1000 alive at 12 years, admissions per child admitted, and in mean LOS. In all other age groups, males ranked above females for these three measures, regardless of whether the child had a birth defect or not.

Newacheck et al. reported information on the extent to which out-of-pocket expenses are financially burdensome for families of children with special health care needs and found these expenses to be three times higher when compared with other children.[24] Our study provides more details of the burden on families of children with a BD with regard to hospital admissions – these children will attend hospital more often and have longer LOS, than for other children of their own age even for admissions not directly due to their BD. This was particularly evident for the children in the younger ages. Waitzman et al.[25] estimated the economic costs of 18 of the most clinically significant birth defects using the California Birth Defects Monitoring Program. However, as the children were not linked to their hospital admissions the study needed to make allowances for "missing discharges" – admissions where their condition might be considered incidental to the primary reason for admission. Our study was able to detect all such admissions for all children registered with a birth defect in WA.

Children with a major BD were 2.4 times more likely to be admitted for nonsuppurative otitis media. Casselbrant et al. outlined various risk factors considered important in the occurrence, recurrence, and persistence of middle-ear disease, including immunocompetence (as in Down syndrome) and craniofacial abnormalities such as cleft palate.[26] Children with Down syndrome also have abnormalities of their Eustachian tube, predisposing them to middle ear infections.[27]

## Conclusion

Whilst it is encouraging that the mean and median LOS in hospital for admissions before the age of 5 years has decreased for all children since 1980, the mean number of admissions per child admitted has remained constant at around 2.2 admissions for children without BDs and 3.8 admissions for children with a BD. Our linked data could be used to investigate further the public health impact of medical care utilisation for children with BDs in Australia, as has been called for in the US.[28] Collection of additional data could assist in ascertaining the reasons for the increase in admissions in children with BDs and whether outcomes have improved. These findings also raise the question of whether there is an opportunity for children with BDs to be monitored and seen earlier in the primary care setting for childhood illnesses not associated with treatment of the BD – to avoid hospitalisation or reduce the LOS.

## Competing interests

The authors declare that they have no competing interests.

## Authors' contributions

Both authors 1) have made substantial contributions to conception and design, or acquisition of data, or analysis and interpretation of data; 2) have been involved in drafting the manuscript or revising it critically for important intellectual content; and 3) have given final approval of the version to be published.

## Authors' information

Clinical Professor Carol Bower is the Medical Specialist for the Western Australian Birth Defects Registry.

## Pre-publication history

The pre-publication history for this paper can be accessed here:


